# Self-Reported High-Cholesterol Prevalence in the Brazilian Population: Analysis of the 2013 National Health Survey

**DOI:** 10.5935/abc.20170055

**Published:** 2017-05

**Authors:** Paulo A. Lotufo, Raul D. Santos, Andrei C. Sposito, Marcelo Bertolami, Jose Rocha-Faria Neto, M. Cristina Izar, Celia Szwarcwald, Rogério R. Prado, Sheila R. Stoppa, Deborah C. Malta, Isabela M. Bensenor

**Affiliations:** 1 Centro de Pesquisa Clínica e Epidemiológica da Universidade de São Paulo, São Paulo, SP - Brazil; 2 Faculdade de Medicina da Universidade de São Paulo, São Paulo, SP - Brazil; 3 Instituto do Coração do Hospital das Clínicas da Faculdade de Medicina da USP, São Paulo, SP - Brazil; 4 Faculdade de Ciências Médicas da Universidade Estadual de Campinas, Campinas, SP - Brazil; 5 Instituto Dante Pazzanese de Cardiologia, São Paulo, SP - Brazil; 6 EpiCenter - Centro de Pesquisa Clínica e Epidemiológica da Escola de Medicina - Pontifícia Universidade Católica do Paraná, Curitiba, PR - Brazil; 7 Escola Paulista de Medicina da Universidade Federal de São Paulo, São Paulo, SP - Brazil; 8 Fundação Instituto Oswaldo Cruz - Instituto de Comunicação e Informação Científica e Tecnológica em Saúde, Rio de Janeiro, RJ - Brazil; 9 Ministério da Saúde - Secretaria de Vigilância em Saúde - Departamento de Doenças e Agravos não Transmissíveis e Promoção da Saúde, Brasília, DF - Brazil; 10 Universidade Federal de Minas Gerais, Belo Horizonte, MG - Brazil

**Keywords:** Cholesterol, Dyslipidemias, Epidemiology, Coronary Artery Disease, Prevalence, Health Survey

## Abstract

**Background:**

Data on the prevalence of dyslipidemia in Brazil are scarce, with surveys available only for some towns.

**Objective:**

To evaluate the prevalence of the self-reported medical diagnosis of high cholesterol in the Brazilian adult population by use of the 2013 National Health Survey data.

**Methods:**

Descriptive study assessing the 2013 National Health Survey data, a household-based epidemiological survey with a nationally representative sample and self-reported information. The sample consisted of 60,202 individuals who reported a medical diagnosis of dyslipidemia. The point prevalence and 95% confidence interval (95%CI) for the medical diagnosis of high cholesterol/triglyceride by gender, age, race/ethnicity, geographic region and educational level were calculated. Adjusted odds ratio was calculated.

**Results:**

Of the 60,202 participants, 14.3% (95%CI=13.7-14.8) never had their cholesterol or triglyceride levels tested, but a higher frequency of women, white individuals, elderly and those with higher educational level had their cholesterol levels tested within the last year. The prevalence of the medical diagnosis of high cholesterol was 12.5% (9.7% in men and 15.1% in women), and women had 60% higher probability of a diagnosis of high cholesterol than men. The frequency of the medical diagnosis of high cholesterol increased up to the age of 59 years, being higher in white individuals or those of Asian heritage, in those with higher educational level and in residents of the Southern and Southeastern regions.

**Conclusion:**

The importance of dyslipidemia awareness in the present Brazilian epidemiological context must be emphasized to guide actions to control and prevent coronary heart disease, the leading cause of death in Brazil and worldwide.

## Introduction

Coronary artery disease or ischemic heart disease is one of the major causes of morbidity and mortality worldwide.^1^ At the beginning of the 1960s, long-term observation studies, mainly the *Framingham Heart Study*, reported that high cholesterol levels doubled the risk for myocardial infarction.^2^ Detailing the cholesterol fractions has allowed identifying low-density lipoprotein cholesterol (LDL-C) as the determinant of the atherogenic process, with a strong and constant association with cardiovascular events.^3^

A meta-analysis with over 170,000 individuals randomized to receive placebo or statins or low versus high doses of those drugs has shown that, for every 40-mg/dL reduction in LDL-C, there was a relative drop of 10%, 20%, 27%, 21% and 25% in all-cause mortality, mortality due to cardiovascular disease, myocardial infarction, ischemic stroke and myocardial revascularization, respectively. There was no beneficial difference regarding the previous presence of cardiovascular disease; however, the absolute benefit was proportional to the previous risk of cardiovascular events, being twice higher in secondary prevention.^4^

Despite the importance of the relation "cholesterol - coronary disease" and the evidence that justifies the control of cholesterol levels at the population level, population surveys conducted in several countries have revealed relatively low rates of diagnosis, knowledge, treatment and control of high cholesterol levels.^5^

The 2013 Brazilian National Health Survey (PNS) is a unique opportunity to estimate the population prevalence of self-reported dyslipidemia in adults (18 years or older), a representative sample of Brazil, its large geographic regions and federated units, urban and rural areas and self-reported educational level and race/ethnicity.

## Methods

### Sample

Descriptive study using 2013 PNS data. The PNS is an epidemiological survey based on households, a representative sample of Brazil, its large regions and federated units, capitals and metropolitan regions. Further information on the PNS can be found in other publications.^6-7^

The minimum sample size was 1,800 households per federated unit, with an initial total sample of 81,767 households planned. In addition, the sample was defined based on the level of accuracy desired for estimation of indicators of interest (proportions of individuals at certain categories). After data collection, registries of the interviews of 64,348 households were obtained, with 60,202 individuals interviewed. The other 4,146 residents selected were excluded because: (i) they refused to answer the specific questionnaire or (ii) had their information rejected in the automatic coherence screening performed by the Brazilian Institute of Geography and Statistics (IBGE). The rate of non-reply was 14.0%.

The PNS sample was designed in three stages. The primary units of sampling (PUS) were the census tracts or sets of sectors. The households were the secondary units, and the adult residents (≥ 18 years) were the tertiary units. Weight factors were calculated for each sample unit, considering the selection probability. The weight for the selected resident was calculated considering the household weight, non-reply adjustments by sex and calibration for the population totals by sex and age groups estimated with the weight of all residents. The PNS is part of the Integrated System of Household Surveys of IBGE, therefore, the PUS considered in this study are a subsample of the set of PUS existing in the master sample of IBGE. The households were selected based on the most recent version, available at the time, of the National Register of Addresses for Statistical Purposes. Details on the sampling process and weighing can be obtained in the previous publication on PNS results.^8^

### Identification of dyslipidemia

At this stage of the survey, the information on the presence or absence of dyslipidemia was obtained via a self-report of the participants based on the results provided by their physicians about the diagnosis of "high cholesterol or triglyceride levels". The first question was *"when did you have the last blood test to measure cholesterol and triglyceride levels"*. The alternatives ranged from *"less than 6 month ago"* to *"more than 3 years ago*" and *"never"*. Only those reporting at least one cholesterol measurement were asked *"has any physician ever diagnosed you with high cholesterol?"* If the answer was positive, the following questions were asked *"how old were you when first diagnosed with high cholesterol?"* and *"has any physician or health care provider given you a recommendation on high cholesterol?".*

### Statistical analysis and ethical procedures

Using the sample base, the point prevalence estimate and 95% confidence interval (95%CI) were calculated for 'the diagnosis of high cholesterol by a physician', 'undergoing a cholesterol test once' and 'measuring cholesterol or triglyceride levels'. The frequencies were stratified by sex, age group (18 to 29 years, 30 to 59, 60 to 64, 65 to 74, and 75 years and older), educational level (none and incomplete elementary school; complete elementary school and incomplete middle school; complete middle school and incomplete high school; and complete high school) and race/ethnicity (white, black and mixed). The prevalence rates were presented for the country, its large geographic regions, federated units and urban and rural areas. The raw frequency for each specific category was presented, as was the odds ratio adjusted for the other variables, except for itself. Data were analyzed with the Stata® software, 11.0 version (Stata Corp., College Station, United States), using the set of commands for data analysis of a complex sample (survey). Statistically significant differences at the 5% level were considered in the absence of 95%CI overlapping.

The PNS was approved by the National Committee on Ethics in Research (CONEP) of the National Board of Health (CNS), of the Brazilian Ministry of Health (protocol n° 328.159, of June 26, 2013). Participation in this study was voluntary and data confidentiality was guaranteed. The adults selected to answer the interview and who agreed to participate in this study signed the written informed consent.

## Results

The PNS estimates discussed in this study are based on the answers of 60,202 individuals aged 18 years and more. The proportion of participants who never had their cholesterol and/or triglyceride levels measured was relatively low, 14.3% (95%CI = 13.7-14.8). Table 1 shows that more than half of the responders had their cholesterol and/or triglyceride levels measured within the past year, with a significantly higher number of women, elderly, white individuals and individuals with complete high educational level, and a lower number of residents of the Northern and Northeastern regions.

**Table 1:** Proportion of participants reporting cholesterol or triglyceride measurement within the past year

Variables	%	95% CI		Raw OR	95% CI		p	Adjusted OR	95% CI		p
		Lower	Upper		Lower	Upper			Lower	Upper	
Brazil	55.4	55.0	55.8								
**Sex**											
Male	48.2	47.3	49.0	1.00				1.00			
Female	61.8	61.3	62.4	1.74	1.69	1.80	< 0.001	1.70	1.65	1.76	< 0.001
**Age**											
18-29 years	41.7	39.6	43.9	1.00				1.00			
30-59 years	56.6	54.5	58.7	1.82	1.75	1.89	< 0.001	1.92	1.84	2.00	< 0.001
60-64 years	67.2	64.8	69.6	2.87	2.65	3.10	< 0.001	3.34	3.08	3.63	< 0.001
65-74 years	74.2	72.1	76.1	4.01	3.73	4.31	< 0.001	5.13	4.74	5.55	< 0.001
≥ 75 years	71.7	70.1	73.4	3.55	3.25	3.88	< 0.001	4.60	4.19	5.06	< 0.001
**Schooling**											
None – incomplete elementary	51.9	50.5	53.3	1.00				1.00			
Complete elementary – incomplete middle	48.7	47.0	50.3	0.88	0.84	0.92	< 0.001	1.25	1.18	1.32	< 0.001
Complete middle - incomplete high	56.0	54.6	57.4	1.18	1.14	1.23	< 0.001	1.68	1.61	1.76	< 0.001
Complete high	72.7	71.7	73.7	2.47	2.33	2.61	< 0.001	2.81	2.64	2.98	< 0.001
**Race/ethnicity**											
White	60.8	54.8	66.6	1.00				1.00			
Black	52.1	45.8	58.3	0.70	0.66	0.74	< 0.001	0.85	0.80	0.90	< 0.001
Mixed	48.6	41.3	56.0	0.61	0.52	0.72	< 0.001	0.53	0.45	0.63	< 0.001
Native	50.0	43.9	56.2	0.65	0.62	0.67	< 0.001	0.85	0.81	0.88	< 0.001
Region	56.9	50.7	62.9	0.85	0.66	1.09	0.201	1.21	0.94	1.57	0.146
**Northern**											
Northeastern	45.9	43.8	47.9	1.00				1.00			
Southeastern	48.1	46.4	49.8	1.09	1.02	1.17	0.008	1.05	0.98	1.12	0.206
Southern	60.9	59.3	62.4	1.84	1.73	1.96	< 0.001	1.50	1.40	1.61	< 0.001
West-Central	57.8	56.0	59.5	1.61	1.50	1.74	< 0.001	1.32	1.22	1.43	< 0.001
Centro-Oeste	53.6	52.2	55.1	1.36	1.26	1.48	< 0.001	1.22	1.11	1.33	< 0.001

OR: odds ratio; 95% CI: 95% confidence interval. Adjustment for the other variables.

Table 2 shows that among those who reported undergoing at least one cholesterol measurement, the prevalence of a medical diagnosis of high cholesterol in the Brazilian population was 12.5% (9.7% for men and 15.1% for women). The women's probability of having a diagnosis of high cholesterol was 60% higher than that of men. The frequency of high cholesterol according to the age group for both sexes increased up to the age of 59 years; from 60 to 74 years, the values were constant, and from the age of 75 years on, the values decreased. The educational level as a socioeconomic indicator showed similar frequencies at the extremes of the category, "no education" and "complete high school". Participants self-reporting black race/ethnicity had significantly lower frequencies of medical diagnosis of high cholesterol. The lowest self-reported rates of high cholesterol were observed in the Northern and West-Central regions.

**Table 2:** Proportion of individuals aged at least 18 years self-reporting a medical diagnosis of high cholesterol

Variables	%	95% CI		Raw OR	95% CI		p	Adjusted OR	95% CI		p
		Lower	Upper		Lower	Upper			Lower	Upper	
Brazil	12.5	12.1	13.0								
**Sex**											
Male	9.7	9.0	10.3	1.00				1.00			
Female	15.1	14.4	15.7	1.66	1.58	1.74	< 0.001	1.61	1.53	1.70	< 0.001
**Age**											
18-29 years	2.8	2.3	3.3	1.00				1.00			
30-59 years	13.3	12.6	13.9	5.26	4.77	5.81	< 0.001	5.04	4.55	5.57	< 0.001
60-64 years	25.9	23.2	28.6	12.02	10.65	13.57	< 0.001	11.13	9.83	12.60	< 0.001
65-74 years	25.5	23.3	27.7	11.76	10.48	13.19	< 0.001	10.79	9.57	12.17	< 0.001
≥ 75 years	20.3	17.7	23.0	8.78	7.69	10.02	< 0.001	7.89	6.87	9.05	< 0.001
**Schooling**											
None – incomplete elementary	15.8	15.0	16.7	1.13	1.05	1.22		0.95	0.88	1.03	
Complete elementary – incomplete middle	10.1	8.9	11.3	0.67	0.61	0.74	0.001	0.90	0.82	0.99	0.191
Complete middle - incomplete high	9.1	8.4	9.8	0.60	0.56	0.65	< 0.001	0.82	0.75	0.89	0.038
Complete high	14.3	12.9	15.6	1.00			< 0.001	1.00			< 0.001
**Race/ethnicity**											
White	13.4	12.6	14.1	1.00				1.00			
Black	11.2	9.7	12.8	0.82	0.75	0.90	< 0.001	0.83	0.76	0.92	< 0.001
Mixed	16.1	11.3	22.5	1.25	0.99	1.57	0.056	1.26	1.00	1.60	0.054
Native	11.8	11.1	12.5	0.87	0.83	0.91	< 0.001	0.98	0.92	1.04	0.441
Region	15.1	11.2	20.1	1.16	0.82	1.63	0.408	1.33	0.93	1.90	0.118
**Northern**											
Northeastern	10.2	9.2	11.1	1.00				1.00			
Southeastern	12.2	11.5	12.9	1.23	1.10	1.37	< 0.001	1.12	1.01	1.26	0.040
Southern	13.3	12.4	14.1	1.35	1.22	1.50	< 0.001	1.15	1.04	1.29	0.010
West-Central	13.0	11.8	14.2	1.32	1.18	1.48	< 0.001	1.14	1.01	1.29	0.037
Centro-Oeste	11.0	10.1	11.9	1.09	0.95	1.25	0.212	1.00	0.87	1.15	0.971

OR: odds ratio; 95% CI: 95% confidence interval. Adjustment for the other variables.

**Figure 1:**
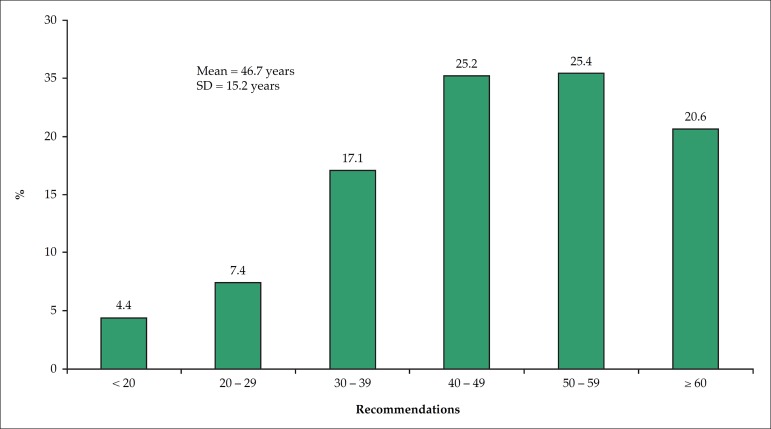
Age at the time of the first diagnosis of high cholesterol levels.

**Figure 2:**
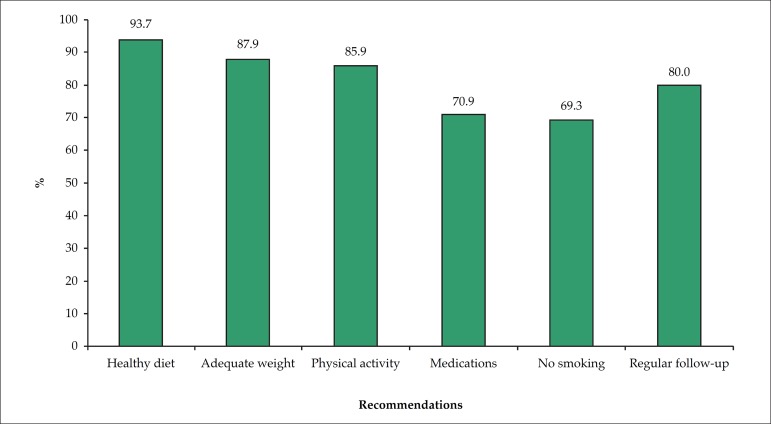
Recommendations to the participants who reported medical diagnosis of high cholesterol.

Figure 1 shows that half of the participants had their first medical diagnosis of high cholesterol in their fifth or sixth decade. One fifth of the interviewees reported having their first medical diagnosis of high cholesterol after the age of 60 years. Figure 2 shows the recommendations provided to individuals in the face of their medical diagnosis of high cholesterol, the most common being "to eat healthy", "to maintain an adequate weight" and "to practice physical activity". Drug prescription was recommended in two-thirds of the cases.

## Discussion

This study shows, for the first time, the prevalence of dyslipidemia in a representative sample of the Brazilian population, in which one in every eight individuals self-reported having high cholesterol levels. The frequency of the self-reported diagnosis was higher among women, white and Asian-heritage individuals, those with higher educational level and residents of the Southern, Southeastern and West-Central regions.

According to the 2014 VIGITEL-BRASIL (Brazilian surveillance system on risk and protective factors of chronic diseases via telephone survey), performed in the 27 Brazilian capitals, the prevalence of a previous medical diagnosis of dyslipidemia was 20.0% (women, 22.2%, and men, 17.6%). In both sexes, the diagnosis of high cholesterol was associated with age increase and higher educational level. Such data are in accordance with the results of this analysis based on the PNS data.^9^

Prevalence studies of the medical diagnosis of high cholesterol have shown low sensitivity for the definitive diagnosis (55%) as shown in the *Third National Health and Nutrition Examination Survey* carried out in the United States (1988-1994) with a representative population of that country.^10^ In a survey involving 12 European countries, self-report associated with only 30% of the cases diagnosed with the specific test.^11^ Despite the low sensitivity, when participants of the *Women´s Health Study* reported their cholesterol level measured, there was strong association between those self-reported values and the higher incidence of cardiovascular disease in 10 years.^12^

The Brazilian Longitudinal Study of Adult Health (ELSA-Brasil) has compared the self-report of high cholesterol medical diagnosis with the diagnosis based on an LDL-cholesterol measurement greater than 130 mg/dL or the use of lipid-lowering drugs. The following were obtained: sensitivity of 51.5% (95%CI = 50.4%-52.5%); specificity of 86.0% (65.1-86.8); and positive and negative likelihood ratios of 3.7 (3.4-3.9) and 0.56 (0.55-0.58), respectively.

With this prevalence of self-reported high cholesterol of 12.5%, the real prevalence of dyslipidemia in the Brazilian population can be estimated as 46.6%.^13^

The 2013 PNS has the limitations of a population-based survey conducted in a country of continental dimensions. However, considering the Brazilian reality, the study design and operation reached an adequate level of quality. Data generalization was relatively safe for the national and regional projections. Surveys, such as the PNS, use self-reported information on medical diagnosis, which is a limited method of assessment. Evaluation by a physician, nurse or via a previously tested standard questionnaire has better accuracy as already proved in a systematic review of population surveys using self-report.^14^ However, considering Brazil's continental dimensions, that is the fastest and most inexpensive way to assess the prevalence of some conditions, such as high cholesterol.

The importance of the PNS data on the medical diagnosis of high cholesterol is justified by the data obtained from a review study of population surveys assessing the historical trend of cholesterol levels since 1980 in three million participants in Latin America, which has concluded that there were few studies on the diagnosis of dyslipidemia in those populations.^5^

The importance of dyslipidemia awareness in the current Brazilian epidemiological context should be emphasized to guide actions of control and prevention of coronary heart disease, the leading cause of death in Brazil and worldwide, and that determines higher mortality in disadvantaged social strata.^15^

## Conclusion

In a representative population sample of Brazil, this study showed that 10% of the men and 15% of the women had a medical diagnosis of high cholesterol.
